# Novel cutting-edge metabolite-based diagnostic tools for aspergillosis

**DOI:** 10.1371/journal.ppat.1006486

**Published:** 2017-09-07

**Authors:** Masha G. Savelieff, Lucia Pappalardo

**Affiliations:** 1 SciGency, Ann Arbor, Michigan, United States of America; 2 Department of Biology, Chemistry and Environmental Sciences, American University of Sharjah, Sharjah, United Arab Emirates; McGill University, CANADA

## Metabolite-based detection of aspergillosis

Aspergillosis is a group of diseases caused by the inhalation of ubiquitous *Aspergillus* spores, generally *Aspergillus fumigatus* [1], that evade the host immune system [2]. Its most aggressive form, invasive aspergillosis (IA), carries a particularly grim prognosis in immunocompromised patients [2]. High mortality and the associated socioeconomic burden [3] necessitate early, accurate, and sensitive detection of *Aspergillus* infection. Many clinical diagnostic tests are available [4]; however, the identification of improved methods for the detection of aspergillosis is still an active field of research.

Pathogens possess a rich metabolism and a vast array of secondary metabolites, many unique to their species, that constitute a pathogen “fingerprint” [5]. Pathogens can leave their imprint on the host in other ways; for instance, host–pathogen interactions can alter the host’s own metabolome, leaving a “signature” of the disease caused by the pathogen [6]. Detecting metabolic evidence of the pathogen’s presence underlies several emerging methods of disease diagnosis. Due to the absence of an ideal diagnostic test, we have seen new applications of metabolite detection for *Aspergillus* in recent years. Methods include detection of gliotoxin [7,8] and siderophores [9], siderophore uptake [10,11], volatile organic compounds (VOCs) [12–14], and changes to the host’s metabolome in serum [15]. Although still at the research stage, all of these techniques offer distinct advantages for aspergillosis detection (Table 1).

**Table 1:** Emerging metabolite-based methods of aspergillosis detection: Advantages and disadvantages of metabolite-based methods of aspergillosis detection at the research stage of development.

Methods of aspergillosis detection	Advantages*	Disadvantages*
**Gliotoxin**^**7**^ **and methylated gliotoxin detection**^**8**^	• Noninvasive • Rapid • Sensitive • Quantifiable • Low sample cost • Has the potential for standardization • Potential detection of actively dividing *Aspergillus* since gliotoxin release is associated with hyphal growth • Amenable to automation	• Not specific to *Aspergillus* • Does not determine infection location • HPLC-MS/MS equipment is costly • Requires sample preparation and metabolite extraction, which increases labor and may introduce contamination • Calibration and addition of internal standard needed to test HPLC-MS/MS performance • The precise relation between fungal metabolite level with time course and severity of infection and IA status according to EORTC/MSG still needs to be determined
**Siderophore detection**^**9**^	• Noninvasive • Rapid • Sensitive • Quantifiable • Low sample cost • Has the potential for standardization • Potential detection of actively dividing *Aspergillus* since TAFC release is associated with nutrient procurement for growth • Potential use for detection in early stages of infection • Amenable to automation	• More specific than gliotoxin but still not specific solely to *Aspergillus* • Does not determine infection location • UPLC-MS/MS equipment is costly • Requires sample preparation and metabolite extraction, which increases labor and may introduce contamination • Calibration and addition of internal standard needed to test instrument performance • The precise relation between fungal metabolite level with time course and severity of infection and IA status still needs to be determined
^**68**^**Ga-labeled siderophores uptake**^**10,11**^	• Noninvasive • Targeted, specifically identifies *Aspergillus* since uptake by other fungi and bacteria was found to be limited • Imaging-based technique, potential to locate the site of infection • Potential detection of actively dividing *Aspergillus* since TAFC release is associated with nutrient procurement for growth • Can differentiate from invading *Aspergillus* versus inert spores	• Sensitivity uncertain • Cross-reactivity still possible with other fungal genera • Requires very specialized radio facilities to produce positron emitter ^68^Ga • Requires very specialized and expensive imaging equipment • Exposes patient to low level of ionizing radiation • The toxicity of TAFC administration to the patient needs to be assessed
**eNose detection of VOCs**^**12,13**^	• Noninvasive • Rapid • Low sample cost • Relatively low equipment cost • Point-of-care testing possible • “Breathprint” profiles or biomarkers have the potential for species identification • Specifically detects disease state, not just the presence of inert *Aspergillus* spores • Has potential to be specific to *Aspergillus*, but sensitivity and specificity are still under investigation	• Does not determine whether infection has spread past the lungs • Contamination from exogenous substances from the air/environment is possible • Confounding parameters still uncertain • Calibration needed to test instrument performance • Initially requires construction of prediction models
**GC-MS detection of VOCs**^**14**^	• Noninvasive • Rapid • Low sample cost • “Breathprint” profiles or biomarkers could enable species identification • Has potential to be specific to *Aspergillus*, but sensitivity and specificity are still under investigation • Amenable to automation	• Does not determine whether infection has spread past the lungs • GC-MS/MS equipment is costly • Contamination from exogenous substances from the air/environment is possible • May require preconcentration of breath samples • Confounding parameters still uncertain • Calibration and addition of internal standard needed to test instrument performance • Initially requires determination of VOCs unique to infecting pathogen
**NMR metabolomics**^**15**^	• Noninvasive • Semi-quantifiable • Low sample cost • Does not require identification of new or unique metabolites specific to *Aspergillus* • Does not require sample manipulation • Amenable to automation	• Low sensitivity • Does not determine site of infection • NMR equipment is costly and requires expensive routine maintenance • Confounding parameters still uncertain • Will initially require construction of prediction models

*Since these methods are still in the research stages of development, some of the stated advantages are only potential advantages, and some of the stated disadvantages may yet be resolved.

**Abbreviations**: ^68^Ga, gallium-68; EORTC-MSG, European Organization for Research and Treatment of Cancer/Invasive Fungal Infections Cooperative Group and the National Institute of Allergy and Infectious Diseases Mycoses Study Group; GC-MS, gas-chromatography mass spectrometry; HPLC-MS/MS; high-performance liquid chromatography tandem mass spectrometry; IA, invasive aspergillosis; NMR, nuclear magnetic resonance; TAFC, triacetylfusarinine C; UPLC-MS/MS, ultra-performance liquid chromatography tandem mass spectrometry; VOCs, volatile organic compounds

## Aspergillosis diagnosis based on metabolite detection or uptake

Gliotoxin is a secondary metabolite secreted during hyphal growth by a number of fungi, including *A*. *fumigatus* [7], and it has been postulated that its detection might coincide with the early stages of infection. Gliotoxin is a potent immune suppressor and may contribute to the failure of the host immune system to prevent opportunistic fungal infections [8]. Gliotoxin has been investigated as a possible biomarker for aspergillosis [16,17], and a comprehensive study employing high-performance liquid chromatography tandem mass spectrometry (HPLC-MS/MS) demonstrated high accuracy and sensitivity for gliotoxin quantification in analytical standards and human serum [7]. Using this accurate method, the authors compared the technique to the galactomannan (GM) assay, a clinical ELISA aspergillosis detection method. Serum samples negative for GM were generally lacking gliotoxin (85%); however, half the GM positive samples were also devoid of gliotoxin [7].

Several scenarios could explain the lack of correlation between positive GM and gliotoxin level: (1) false GM positives, (2) decline in gliotoxin at late stages of infection when hyphal growth might slow as fungal burden increases, or (3) chemical instability of the gliotoxin disulfide in vivo [8]. The study did not classify cases of IA according to European Organization for Research and Treatment of Cancer/Invasive Fungal Infections Cooperative Group and the National Institute of Allergy and Infectious Diseases Mycoses Study Group (EORTC/MSG) definitions, so it is not possible to ascertain the degree to which false positive GM assays may have influenced the results of this study. Accurate IA status and duration of illness are also required to determine whether gliotoxin levels correlate with early infection versus late infection, and this was similarly not reported. Therefore, while the accuracy and sensitivity of the test is promising, an in-depth analysis with IA status and time frame will be required to evaluate the precise relation of gliotoxin to IA diagnosis.

To address the possibility of in vivo chemical instability of gliotoxin, levels of an inactive methylated metabolite of gliotoxin, bis(methylthio)gliotoxin (bmGT), were assayed in patient sera by high-performance thin-layer chromatography (HPTLC). These studies indicated that bmGT may be a better metabolic biomarker than gliotoxin, possibly due to its greater stability [8]. In this study, the IA status of patients was assessed according to EORTC/MSG criteria by clinicians who were unaware of the results of the gliotoxin and bmGT assays. Then, prediction of IA status by the GM assay was compared to prediction by bmGT quantification. Employing bmGT quantification had greater sensitivity and positive predictive value for IA than GM, and, combined, the 2 diagnostic tests identified all positive IA cases and almost totally avoided false negatives [8]. This study incorporated EORTC/MSG definitions of IA, strengthening the clinical applicability of these findings compared to the findings of the earlier gliotoxin study [7].

Siderophores are secondary metabolites utilized by microorganisms to scavenge the micronutrient iron, necessary for growth, from the environment or host [9]. Therefore, their presence implies an actively dividing pathogen. Triacetylfusarinine C (TAFC) is produced by a few fungal genera, *Aspergillus* amongst them, with no known human function, so it is neither anticipated in healthy hosts [9] nor expected to be taken up by host cells [10]. TAFC in serum from patients at risk of IA can be quantified by ultra-performance liquid chromatography tandem mass spectrometry (UPLC-MS/MS) [9]. Fifty-eight suspected, probable, or proven IA cases classified according to EORTC criteria had serum assayed for GM (≥0.5 index threshold) and TAFC. A positive correlation was observed between GM score and TAFC (Pearson *r* value = 0.77), but, interestingly, TAFC levels above the threshold of detection were observed more frequently in suspected IA than in probable/proven IA, possibly because the latter received antifungals [9]. Furthermore, a subset of samples from patients with suspected IA were TAFC positive but GM negative, suggesting TAFC secretion may occur early during infection [9]; however, the GM results may also have been false negatives, and further work is required to clarify this.

The analytical methods for metabolite (gliotoxin, bmGT, and TAFC) quantification are accurate and sensitive, but they require further research to uncover the true correlation between serum level with IA status. These studies will require evaluating the time course of metabolite production during the natural history of the disease and in patients with a range of severity of infection. Use of EORTC/MSG definitions of disease will be critical to allow comparison of the results of these studies with those evaluating other diagnostic tests.

TAFC can bind the positron emitter gallium-68 (^68^Ga) creating ^68^Ga-TAFC complexes for positron emission tomography (PET) [10]. Biodistribution studies in healthy mice demonstrated rapid renal ^68^Ga-TAFC elimination, suggesting it was not actively taken up in healthy animals [10]. This was confirmed in Lewis rats in which ^68^Ga-TAFC uptake in the lungs was selective in rats with IA and not observed in healthy rats and *Aspergillus*-challenged rats that did not develop IA [10]. ^68^Ga-TAFC uptake was dependent on infection severity, suggesting deliberate uptake by *Aspergillus*.

*Aspergillus*-challenged rats that did not develop IA did not absorb ^68^Ga-TAFC, implying that the technique differentiates between actively growing, invading *Aspergillus* compared to exogenous, inert spores inhabiting the airways [10]. This observation also suggests that the method could detect IA early in the course of infection when *Aspergillus* is in a phase of rapid growth and prior to the development of a high burden of fungal disease [10]. In addition, whole body scans can reveal infection spread to organs other than lungs. ^68^Ga-TAFC uptake by *Aspergillus* was greater than by other fungi and almost nonexistent by other microorganisms, indicating it could be specific for *Aspergillus* [11]. The high uptake and retention time of ^68^Ga-TAFC by *Aspergillus* enhances sensitivity [10] but lengthens the duration patients would be exposed to radiation, a disadvantage of the technique. The application of gallium-68 complexes in nuclear medicine is well documented [18]; however, the safety of gallium-68 with TAFC in particular needs to be evaluated, but studies thus far indicate that ^68^Ga-TAFC uptake may be a useful diagnostic tool for IA.

## Aspergillosis diagnosis based on VOCs

*Aspergillus* produces several volatile metabolites, which can be present in the breath of patients with lung infection [19]. Detection of these VOCs is an attractive strategy for the detection of IA, since breath collection is noninvasive and desirable for critically ill patients susceptible to IA. Earlier attempts to detect VOCs focused on the detection of single metabolites such as 2-pentylfuran, which was elevated in patients with *Aspergillus* airway colonization [20]. However, 2-pentylfuran is not unique to *Aspergillus*, and confounding factors affected diagnosis based solely on this VOC [21]. Therefore, more recent methods have included the detection of several VOCs or entire mixtures.

eNoses are affordable, portable options for VOC detection, possibly making point-of-care medical diagnosis a reality [22]. Numerous commercial eNoses exist that vary in the sensor material in order to make them suitable for different applications [23,24]. eNoses contain sensor arrays whose physical properties change upon adsorption of volatiles, e.g., electrical resistance, which is recorded. Volatile metabolites from a sample bind to the sensor arrays, and the combination of these volatiles will produce a pattern of response that reflects this mix of metabolites [23,24]. This response pattern is unique to that particular VOC mixture and forms the basis of detection.

The feasibility of eNose-based detection of aspergillosis was examined in patients with prolonged chemotherapy-induced neutropenia (PCIN) [12] and cystic fibrosis (CF) [13]. PCIN patients underwent full diagnostic workup for aspergillosis (classified by EORTC criteria) whilst CF patients were diagnosed by sputum culture. Each patient’s breath was analyzed by a Cyranose 320 eNose to produce a “breathprint,” a variation in the electrical resistance differential across all 32 eNose sensors. All “breathprint” data from uninfected controls and patients with proven and probable IA were subjected to principal component analysis, identifying signal components that accounted for 99.9% variance between control and experiment. These components were used to construct prediction models, which had cross-validation of about 89% or higher [12,13].

The eNose’s ability to detect aspergillosis in the presence of 2 underlying medical conditions (PCIN and CF) demonstrates its broad applicability [12,13]. Early eNose detection of VOCs from the host inflammatory response to infection may allow for the detection of infection prior to the development of significant fungal disease. It may even be possible to make eNose detection specific for *Aspergillus* since breathprints of CF patients with lung coinfections by other microorganisms were distinct from breathprints from patients with aspergillosis [13], as supported by in vitro tests [25]. The main drawback of eNose technology is that prior analysis is required to establish the prediction model, and a reliable calibration system is required to ensure the same operation between different eNose machines. These challenges will have to be addressed before eNose technology can become a widely used standardized diagnostic technique.

Gas-chromatography mass spectrometry (GC-MS) is another method that can be used to identify volatile metabolites based on their mass spectrometry (MS) profile. Combined with patient breath collection, it is noninvasive and can structurally identify metabolites in “disease breath.” GC-MS was used to identify secondary metabolite mixtures unique to *A*. *fumigatus* compared to other aspergilli [14]. The combination of monoterpenes (camphene, α-pinene, β-pinene, limonene) and sesquiterpenes (α-trans-bergamotene, β-trans-bergamotene) was found to be specific to *A*. *fumigatus* in vitro [14]. This observation was recapitulated in IA patient breath samples, which additionally contained 2 metabolites not detected in vitro, the terpenoid ketone trans-geranylacetone and a β-vatirenene–like sesquiterpene [14].

The IA status of 64 enrolled patients was evaluated independently by 2 doctors as “proven,” “probable,” or “possible” according to EORTC/MSG criteria. Breath VOCs were submitted to GC-MS and analyzed by heat map for the 8 metabolites found to define *A*. *fumigatus*. IA status of 60/64 cases were correctly identified by this GC-MS analysis, highlighting the potential of this technique for IA diagnosis [14]. GC-MS of patient breath is noninvasive and can be tailored to detect other invasive pathogens to extend its utility to the diagnosis of other diseases. That would require preliminary in vitro work to discover secondary metabolites or combinations unique to a pathogen/strain followed by validation in patient breath samples. This requires more labor and doesn't guarantee that findings in vitro translate to patient samples. In addition, it may not be possible to discover a combination of metabolites unique to the pathogen, which is the principal drawback of this method.

## Aspergillosis diagnosis based on metabolomics

Metabolomics is the untargeted, system-wide detection of metabolites in biological samples and has great potential for clinical use. It may be employed to either (1) detect metabolite mixtures unique to infecting pathogens or (2) observe changes to the host’s metabolome caused by infection [26]. Typically, metabolite detection is by MS or nuclear magnetic resonance (NMR), followed by statistical analysis to reveal system-wide differences in all systematically varying metabolites between healthy versus infected patients [26].

NMR metabolomics is well suited to study biofluids in their native state and was applied to aspergillosis detection in falcons (gyrfalcons and gyr-x peregrine hybrids) [15]. Blood samples were withdrawn from clinically healthy falcons and falcons with confirmed aspergillosis and analyzed by 1D ^1^H-NMR spectroscopy. Multivariate statistical analysis of all identified host metabolites from the resultant spectra showed a clear metabolic separation of healthy versus diseased falcons, each with a distinct metabolic profile [15]. Additionally, because NMR metabolomics can identify metabolites, information about which contributed to the distinction between healthy versus diseased cohorts was revealed. In particular, 3-hydroxybutyrate was significantly elevated in the blood of aspergillosis falcons compared to healthy raptors [15].

NMR metabolomics produces a top-down systematic cataloging of host metabolites that vary consistently between infected and healthy patients [26]. Unlike other diagnostic methods that detect *Aspergillus* or its components, NMR metabolomics produces a host “disease metabolic profile” of aspergillosis. This indicator of disease differentiates from the detection of exogenous *Aspergillus* spores by some methods that could give false positives [27]. The promising, preliminary study in falcons suggests that ^1^H-NMR metabolomics could translate to humans as a detection tool for aspergillosis.

Despite the advantages of the systems-wide approach by NMR metabolomics, it suffers from low sensitivity and resolution. While the more abundant, primary metabolites may be readily identified, less abundant and more structurally complex secondary metabolites are more challenging to identify, although advances in data analysis are constantly being made.

## Aspergillosis detection and beyond: Where else can metabolite detection take us?

Several metabolite-based methods have shown promise for improved *Aspergillus* detection, which satisfy the criteria of early detection, low invasiveness, low cost, and point-of-care [7–15]. The timely detection of aspergillosis will significantly facilitate the decision to treat infected immunocompromised patients—to improve their prognosis for recovery whilst also preventing unnecessary prophylactic administration of potentially toxic antifungals.

Beyond *Aspergillus* detection, metabolite identification and quantification in addition to the more recent system-wide metabolomics methods may be extended to other microbial and viral infections. Metabolomics can address questions pertaining to infection mechanisms and host–pathogen interactions [28]. These studies may reveal pathogen vulnerabilities that may be exploited to develop therapeutic strategies. Therefore, in addition to diagnosis, metabolomics may be applied in drug design, an essential area of research in this era of mounting microbial resistance [29]. The promise of metabolomics is only now unfolding, and its utility is being recognized in various research areas with novel potential future applications.
